# Reducing Pup Litter Size Alters Early Postnatal Calcium Homeostasis and Programs Adverse Adult Cardiovascular and Bone Health in Male Rats

**DOI:** 10.3390/nu11010118

**Published:** 2019-01-08

**Authors:** Jessica F. Briffa, Rachael O’Dowd, Tania Romano, Beverly S. Muhlhausler, Karen M. Moritz, Mary E. Wlodek

**Affiliations:** 1 Department of Physiology, The University of Melbourne, Parkville 3010, Australia; jessica.griffith@unimelb.edu.au (J.F.B.); odowd.rachael.a@edumail.vic.gov.au (R.O.); t.romano@latrobe.edu.au (T.R.); 2 Department of Physiology, Anatomy and Microbiology, LaTrobe University, Bundoora 3083, Australia; 3 Department of Food and Wine Science, School of Agriculture, Food and Wine, FOODplus Research Centre, The University of Adelaide, Adelaide 5064, Australia; Beverly.Muhlhausler@adelaide.edu.au; 4 Child Health Research Centre and School of Biomedical Sciences, The University of Queensland, St. Lucia 4101, Australia; k.moritz1@uq.edu.au

**Keywords:** reduced litter size, postnatal calcium homeostasis, adult bone health, milk composition

## Abstract

The in utero and early postnatal environments play essential roles in offspring growth and development. Standardizing or reducing pup litter size can independently compromise long-term health likely due to altered milk quality, thus limiting translational potential. This study investigated the effect reducing litter size has on milk quality and offspring outcomes. On gestation day 18, dams underwent sham or bilateral uterine vessel ligation surgery to generate dams with normal (Control) and altered (Restricted) milk quality/composition. At birth, pups were cross-fostered onto separate dams with either an unadjusted or reduced litter size. Plasma parathyroid hormone-related protein was increased in Reduced litter pups, whereas ionic calcium and total body calcium were decreased. These data suggest Reduced litter pups have dysregulated calcium homeostasis in early postnatal life, which may impair bone mineralization decreasing adult bone bending strength. Dams suckling Reduced litter pups had increased milk long-chain monounsaturated fatty acid and omega-3 docosahexaenoic acid. Reduced litter pups suckled by Normal milk quality/composition dams had increased milk omega-6 linoleic and arachidonic acids. Reduced litter male adult offspring had elevated blood pressure. This study highlights care must be taken when interpreting data from research that alters litter size as it may mask subtle cardiometabolic health effects.

## 1. Introduction

It is well known that the in utero and early postnatal environments play crucial roles in offspring growth, development and long-term health. David Barker first demonstrated a causal link between size at birth and later-life cardiovascular disease [1,2], which has been since expanded to include several adverse pregnancy perturbations, including fetal growth restriction [3,4], maternal undernutrition [5,6], maternal alcohol consumption [7,8] and maternal stress [9,10]. Postnatal growth rate and development is directly proportional to the quality and quantity of milk produced and is particularly influenced by fatty acid composition [11]. Specifically, high intakes of omega-3 fatty acids in early postnatal life is associated with reduced fat deposition and improved cardiometabolic health [12,13].

More recent experimental studies have demonstrated that altered maternal nutrition during the lactation period can program adult offspring cardiometabolic disease. Specifically, pups suckled by dams fed a cafeteria diet during lactation exhibit a “thin-outside-fat-inside phenotype” (lean with increased abdominal fat) and impaired metabolic health in adulthood [14]. Whereas, male offspring cross-fostered onto a dam fed an isocaloric low-protein diet (6% protein) throughout pregnancy and lactation have increased blood pressure and renal dysfunction [15,16]. As milk is the sole source of nutrition during early postnatal life, these studies strongly support a role for altered milk quality and/or quantity as a mechanism through which maternal nutritional status during lactation influences offspring disease.

In the developmental programming field, many researchers standardize or reduce litter size at birth to normalize milk intake across pups and cohorts [17], with the degree of litter size reduction dependent on the experimental model and research question [18,19,20,21,22,23,24,25]. A previous study identified that pup body weight and development during lactation is dependent on litter size at birth [26], which the authors hypothesize is likely due to mutual maternal and offspring adjustment to a genetically determined litter size [26]. This suggests that studies that cull pups to standardize litter size have the potential to disturb this biological process and that the original litter size may continue to have some influence on offspring development. In line with this, we previously demonstrated that reducing the litter size of healthy Wistar Kyoto (WKY) rat dams at birth (from 10–14 pups per litter to 5 pups per litter) decreases offspring body weight during early life and increases adult male blood pressure likely due to mesenteric artery stiffness and compromises bone health [27,28,29,30,31]. Whereas, severely reducing litter size to 3 pups (inducing early postnatal overnutrition) increases offspring body weight by weaning, alters cardiac structure and function and programs poor metabolic health [32,33]. Thus, there is a need to better understand the impact of reducing litter sizes to differing extents on the subsequent outcomes of the pups.

The mechanism behind this disease programming due to reducing litter size is likely due to its effect on milk composition. Specifically, we have recently demonstrated that both the dam and pup can modulate the maternal milk composition [34]. Thus, dams suckling growth restricted pups have improved milk fatty acid composition (characterized by increased LC-polyunsaturated fatty acid (PUFA) and LC-monounsaturated fatty acid (MUFA)), which is likely to be a compensatory mechanism aimed at supporting pup growth and organ development [34]. This finding suggests that the decreased pup milk intake, rather than poor milk quality, results in the aforementioned long-term disease [27,28,29]. Therefore, it is possible that reducing litter size at birth to 5 pups (similarly to the reduction in litter size we observe following uteroplacental insufficiency surgery) can itself induce changes in the early postnatal environment that has consequences for adult health outcomes and may thus mask or exacerbate any offspring outcomes due to adverse pregnancy and/or lactational events.

Therefore, the aim of this study was to investigate the effect of reducing litter size at birth on pup postnatal growth and development, mammary development and maternal milk composition. We additionally characterized if there were sex-specific differences in long-term cardiometabolic and bone health outcomes due to reducing litter size at birth and whether these outcomes were exacerbated if the pup was suckled by a dam with altered milk quality/composition.

## 2. Materials and Methods

### 2.1. Animals

**Figure 1:**
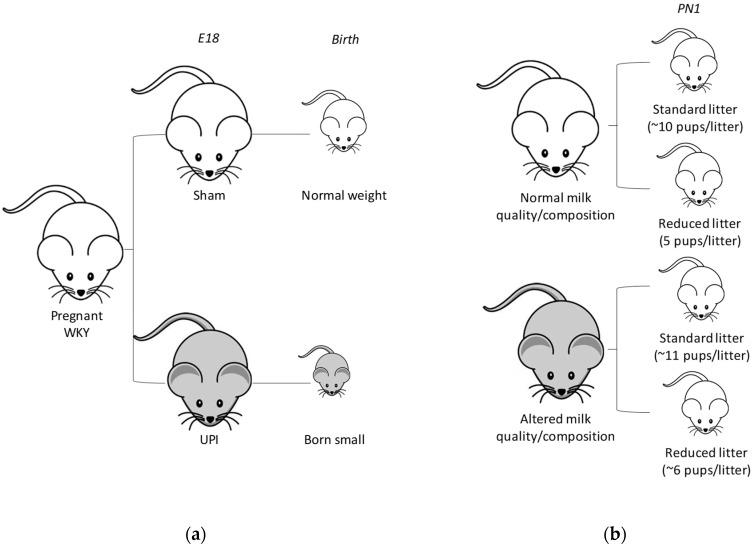
Study design. (**a**) Pregnant Wistar Kyoto (WKY) rats underwent Sham (white) or uteroplacental insufficiency (UPI; grey) surgery on day 18 of gestation (E18) and allowed to deliver naturally at birth. (**b**) On postnatal day 1 (PN1) pups from the Sham surgery dams were cross-fostered onto separate Sham (with Normal milk quality/composition) or UPI (with Altered milk quality/composition) dams with either an intact or reduced litter size.

All experiments were approved by The University of Melbourne’s animal experimentation ethics sub-committee (AEC: 02081) following the National Health and Medical Research Councils (NHMRC) Australian code for the care and use of animals for scientific purposes. Female WKY rats (9 to 13 weeks of age) were obtained from the Animal Resources Centre (Canning Vale, WA, Australia) and provided with a 12-h light/dark cycle at 19–22 °C with ad libitum access to food and water. To generate rats with ‘normal’ and ‘altered’ milk quality/composition, rats were mated and surgery performed on day 18 of gestation (term = 22 days) as described previously [35]. Briefly, F0 pregnant rats were randomly allocated to a sham (Normal milk quality/composition; Control; *n* = 7–8 per group) or uteroplacental insufficiency (Altered milk quality/composition; Restricted; *n* = 8 per group) group, that experience premature lactogenesis [30], and were anaesthetized with 4% isoflurane and 650 mL/min oxygen flow (reduced to 3.2% isoflurane and 250 mL/min oxygen flow when suturing to aid in the animals recovery) to reduce the duration of anesthetic exposure and aid in recovery [35]. Rats were then allowed to deliver naturally (Figure 1a). Pups from the sham (Control) operated dams were cross-fostered 1 day after birth (PN1) randomly onto separate dams (Control or Restricted) where the litter size was unaltered from the surrogate (Standard) or reduced to 5 pups (Reduced) giving rise to four experimental groups (Figure 1b); Standard litter size suckled by Normal milk quality/composition dams (mean litter size 10.38; range 9–12 pups), Reduced litter size suckled by Normal milk quality/composition (mean litter size 5.00; range 5 pups), Standard litter size suckled by Altered milk quality/composition dams (mean litter size 11.50; range 7–15 pups) and Reduced litter size suckled by Altered milk quality/composition (mean litter size 6.00; range 3–9 pups) with *n* = 15–18 dams per group [17,31,36,37,38]. Birth weight (3.5 ± 0.4 g vs. 4.0 ± 0.04 g for Restricted and Control dams, respectively) and litter size (8.5 ± 0.4 pups vs. 11.1 ± 0.4 pups for Restricted and Control dams, respectively) of pups born to ligation surgery (Restricted; Altered milk quality/composition) dams was reduced compared to sham-operated (Control; Normal milk quality/composition) dams.

### 2.2. Study 1: Postnatal Study

On the morning of PN6, one cohort of pups were removed from their dam and weighed, killed via decapitation, and blood was collected and pooled within litters. Aprotinin (Sigma-Aldrich; Castle Hill, NSW, Australia) was added to the tubes used for plasma PTHrP analysis [39]. One pup per litter was frozen whole and stored at −20 °C for whole body calcium analysis. Pup stomach contents were collected for fatty acid analysis. Dams were anaesthetized (Ketamine (50 mg/kg; Parnell Laboratories, Alexandria, NSW, Australia) and Ilium Xylazil-20 (10 mg/kg; Troy Laboratories, Glendenning, NSW, Australia)) 4–6 h after removal of the pup to allow milk to accumulate and milk was collected following gentle massage of the left mammary gland and teats without the need for hormonal stimulation [40], then euthanized by cardiac puncture with blood collected for subsequent analysis. The mammary glands were dissected, weighed and the right mammary gland immediately snap frozen in liquid nitrogen or fixed in 10% neutral buffered formalin (Perrigo; Balcatta, WA, Australia) for histological analysis.

#### 2.2.1. Mammary ‘Real-Time’ PCR and Histology

‘Real-time’ PCR was used to quantitate milk protein gene expression in mammary tissue on PN6 as described previously with *n* = 3–7 dams per group [30]. Briefly, RNA was extracted from mammary tissue using the Polytron PT 3100 (Biolab; Clayton, VIC, Australia) homogenizer and a commercially available kit (RNeasy Lipid Tissue Mini Kit from Qiagen; Chadstone, VIC, Australia). RNA was then DNase treated using the Ambion DNA free kit (Life Technologies; Mulgrave, VIC, Australia). First strand cDNA was generated from 1 µg RNA using the Superscript II Single Stranded cDNA kit (Life Technologies). qPCR was performed then conducted against the milk protein genes *Pthrp*, *b-casein* and *a-lactalbumin* with Ribosomal *18S* as the reference gene [34]. The reaction was activated by heating the mixture to 95 °C for 10 min, then ‘Real-time’ PCR reactions were ran for 40 cycles of 95 °C for 15 s and 60 °C for 60 s. For relative quantification of gene expression, a multiplex comparative threshold cycle method was employed [35]. *18S* values were not different between treatments.

Fixed mammary tissue was processed into paraffin blocks, sectioned at 5 µm and stained with hematoxylin and eosin (*n* = 4–5 per group). Five sections per sample were analyzed for alveolar area and number using ImagePro Software (Medai Cybernetics; Warrendale, PA, United States of America) [40].

#### 2.2.2. PTHrP, Corticosterone, Calcium and Electrolyte Measurements

Plasma, milk and mammary tissue concentrations of PTHrP were quantified by a N-terminal radioimmunoassay with a minimum detection limit of 2 pmol/L, and intra- and inter-assay coefficients of variation of 4.8% and 13.6% respectively with *n* = 7–9 per group [41]. Plasma corticosterone was measured by enzyme immunoassay validated for direct measurements in diluted plasma following the manufacturer’s protocol (Cayman Chemical; Ann Arbor, MI, United States of America) with a minimum detection limit of 30 pg/mL, and intra- and inter-assay coefficients of variation of 7.4% and 7.0% respectively (*n* = 4–8 per group). Total calcium concentrations were determined using colorimetric spectrometry using the Synchron CX-5 Clinical System (Beckman Coulter; Lane Cove, NSW, Australia) and ionic calcium (active or free calcium; regulated by PTHrP), sodium and potassium concentrations were determined using ion selective electrodes correcting for pH (Ciba-Corning model 644; Cambridge, MA, United States of America) from milk as well as from pup and maternal plasma (*n* = 5–8 per group) [39,42]. Total calcium concentration in the pup body was determined after ashing using the CX-5 Analyzer (*n* = 7–9 per group) [30,40]. Total protein and lactose concentrations were analyzed as described previously with *n* = 4–6 per group [30].

#### 2.2.3. Fatty Acid Analysis

Total fatty acid composition of the milk was determined by the direct trans-esterification method of Lepage and Roy [43] as previously described [34] with *n* = 6–7 dams per group. Briefly, 50 to 100 µL of milk was placed into a screw-capped Teflon-lined tube containing C23:0 as an internal standard. After the 1-h trans-esterification procedure and recovery of the fatty acid methyl esters (FAMEs) in the benzene phase, the FAMEs were analyzed by capillary gas liquid chromatography. FAMEs were then separated and measured on a Shimadzu (17A) gas chromatograph with flame ionization detection. A 50 mm × 0.25 mm BPX-70 fused silica capillary column (SGE Scientific; Ringwood, VIC, Australia) with a film thickness of 0.25 µm was used in conjunction with a Shimadzu on-column auto-injector. Ultrahigh purity hydrogen was used as a carrier gas at a flow rate of 2 mL/min. A temperature gradient program was used with an initial temperature of 170 °C, increasing at 3 °C/min to 218 °C. Identification of the FAMEs was made by comparison with the retention times of chromatography reference standard mixtures (Nu-Chek Prep; Elysian, MN, United States of America) [44].

### 2.3. Study 2: Lactation and Adult Study

Another cohort of pups stayed with their cross-fostered dam until weaning (PN35) and were weighed from birth (PN1) to weaning (PN3, PN6, PN10, PN14, PN17, PN21, PN24, PN28 and PN35) to determine pup growth and milk intake. Pups were weighed during a 1-h maternal separation period followed by a 3-h re-feeding period. Milk intake was calculated as a percentage of pup body weight gain after feeding compared to pre-feeding [30] and the area under the milk intake curve was calculated between PN3 and PN17 as an index of milk consumption. After weaning, male and female pups were housed separately. At 6 months, offspring (1 male and 1 female per litter) underwent an intra-arterial glucose tolerance test (IAGTT) and tail-cuff blood pressure measurements as previously described [36,37,38]. Rats were then weighed and anaesthetized with an intraperitoneal injection (50 mg/kg Ketamine and 10 mg/kg Ilium Xylazil-20) and the right hind limbs were collected; all females underwent post-mortem when they were in estrous.

#### 2.3.1. Bone Analyses

The right hind limb was dissected to separate the femur bone from soft tissue and femur length measured using digital calipers (*n* = 9–19 per group per sex). Individual femurs then underwent peripheral quantitative computed tomography (pQCT) to measure bone volumetric content, density and stress strain index using methods previously described [31,45,46,47]. Briefly, two slices of 1 mm thickness (voxel size 0.1000 mm^3^, peel mode 20, contour mode 1) were taken at distances of 15% and 50% from the reference line to quantify both trabecular and cortical bone tissue, respectively. A tissue density of 280 mg/cm^3^ or less was identified as trabecular bone, whereas a density of 710 mg/cm^3^ was representative of cortical bone. Automatic density thresholding (400 mg/cm^3^) was used to eliminate the effects of any soft tissue which may have remained on the femur after dissection. Bone mineral content, density, and stress strain index (index of bone bending strength) were then measured.

#### 2.3.2. Plasma Analyses

Plasma were analyzed for glucose using enzymatic fluorometric analysis and insulin using a rat insulin radioimmunoassay kit (Merck Millipore; Bayswater, VIC, Australia) as previously described (*n* = 6–11 per group per sex) [38,48]. Fasting plasma glucose and insulin was taken as the average of two-time points (10 and 5 min before injection). First-phase insulin secretion was calculated as the incremental area under the insulin curve between 0 and 5 min after the intra-arterial injection of glucose. Homeostasis model assessment for insulin resistance (HOMA-IR) was then determined [38].

### 2.4. Statistical Analysis

Data were analyzed using a two-way ANOVA to determine differences between Pup (Standard or Reduced litter size) and Dam (Normal or Altered milk quality/composition) groups. If an interaction was present in the two-way ANOVA, a Tukey’s post-hoc test was performed to identify any effect of reducing litter size within each maternal group and any effect altering maternal milk quality/composition has on pup outcomes based on litter size. Where appropriate data analysis was performed on each sex. ANOVA statistical analysis was performed using SPSS Statistics 22 (IBM; St Leondards, NSW, Australia). All data are presented as mean ± SEM and a *p* < 0.05 was assessed as being statistically significant.

## 3. Results

### 3.1. Growth to Weaning

As mentioned previously, dams that underwent uteroplacental insufficiency surgery gave birth to pups that were growth restricted. Not surprisingly, however, birth weight in the four experimental groups was not different (as only offspring from Sham-operated dams were utilized in the study) and litter size was decreased in the groups that had their litter size Reduced at birth on PN1 and PN6, with no differences in cannibalism across groups by PN6 (Table 1). From PN6 male (all ages) and female (except for PN14, PN21 and PN28) body weight was decreased (−6% and −7% for males and females, respectively) if they were suckled by a dam with Altered milk quality/composition. Body weight at weaning was not, however, different between groups (PN35; Table 1).

### 3.2. Mammary Structure and Maternal Plasma Analysis

**Figure 2:**
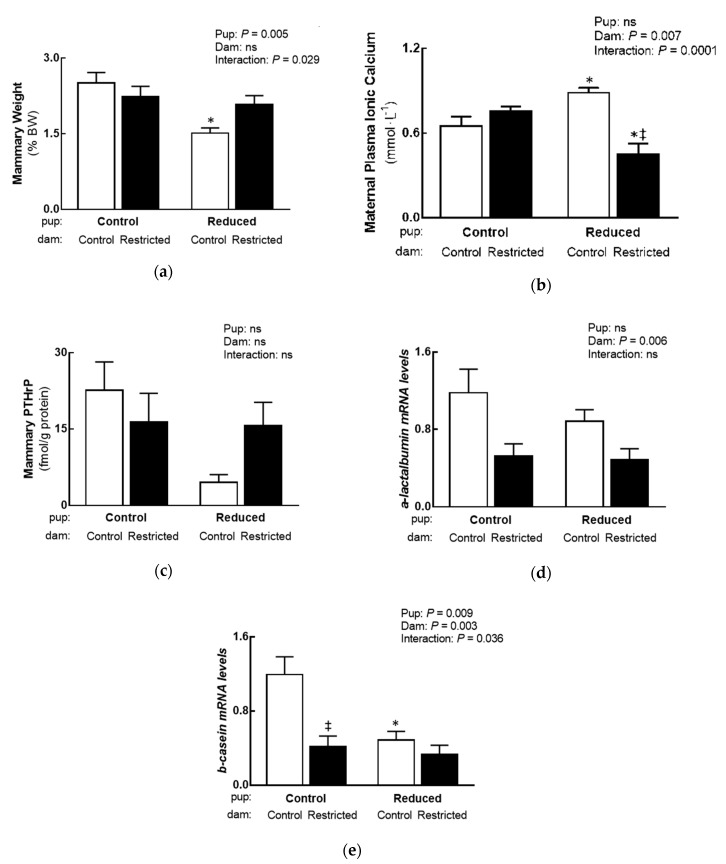
Effect reducing litter size has on maternal and mammary outcomes (*n* = 5–9 per group). (**a**) Mammary weight, (**b**) maternal ionic calcium concentrations and (**c**) mammary PTHrP protein concentration, (**d**) *a-lactalbumin* and (**e**) *b-casein* mRNA expression. Data are analysed with a two-way ANOVA reporting differences between Pup (Standard and Reduced litters) and Dam (Normal and Altered milk quality/composition) groups, with a Tukey’s post-hoc test used to identify where interactions lie. Data presented as the mean ± SEM, where ns is not significant. Significant differences between Standard and Reduced litter pups are indicated by an asterisk (* *p* < 0.05) and differences between sham operated (Control; Normal milk quality/composition) and uteroplacental insufficiency surgery (Restricted; altered milk quality/composition) dams are indicated with a double dagger (‡ *p* < 0.05). Normal milk quality/composition dams denoted by white open bars and Altered milk quality/composition dams denoted by black closed bars.

An interaction between Dam and Pup was identified in mammary weight, where it was decreased in Reduced litter size pups suckled by Normal milk quality/composition dams compared to Standard litter size pups suckled by Normal milk quality/composition dams (−40%, Figure 2a; Tukey’s post-hoc). There was, however, no difference in mammary weight between Standard litter size pups suckled by Altered or Normal milk quality/composition dams; likely due to an increased alveolar number (+25%) in Altered milk quality/composition dams, but not area (Table 2). There were no differences in maternal plasma PTHrP and total calcium between groups (Table 2). An interaction between Dam and Pup was identified in maternal ionic calcium, whereby it was increased in Reduced litter size pups suckled by Normal milk quality/composition dams compared to Standard litter size pups suckled by Normal milk quality/composition dams (+36%) and Reduced litter size pups suckled by Altered milk quality/composition dams (+97%), but was decreased in Reduced litter size pups suckled by Altered milk quality/composition dams compared to Standard litter size pups suckled by Altered milk quality/composition dams (−40%; Figure 2b; Tukey’s post-hoc). Maternal corticosterone (Table 2) along with mammary PTHrP (mRNA and protein; Table 2 and Figure 2c) were not affected by litter size or maternal treatment. A main Dam effect was identified in mammary *a-lactalbumin* gene abundance, where it was decreased in Altered milk quality/composition dams (−51%; Figure 2d). An interaction between Dam and Pup was identified in mammary *b-casein*, which was lower in Standard litter size pups suckled by Altered milk quality/composition dams (−65%) and Reduced litter size pups suckled by Normal milk quality/composition dams (−59%) compared to Standard litter size pups suckled by Normal milk quality/composition dams (Figure 2e; Tukey’s post-hoc).

### 3.3. Pup Plasma Analysis and Milk Consumption

**Figure 3:**
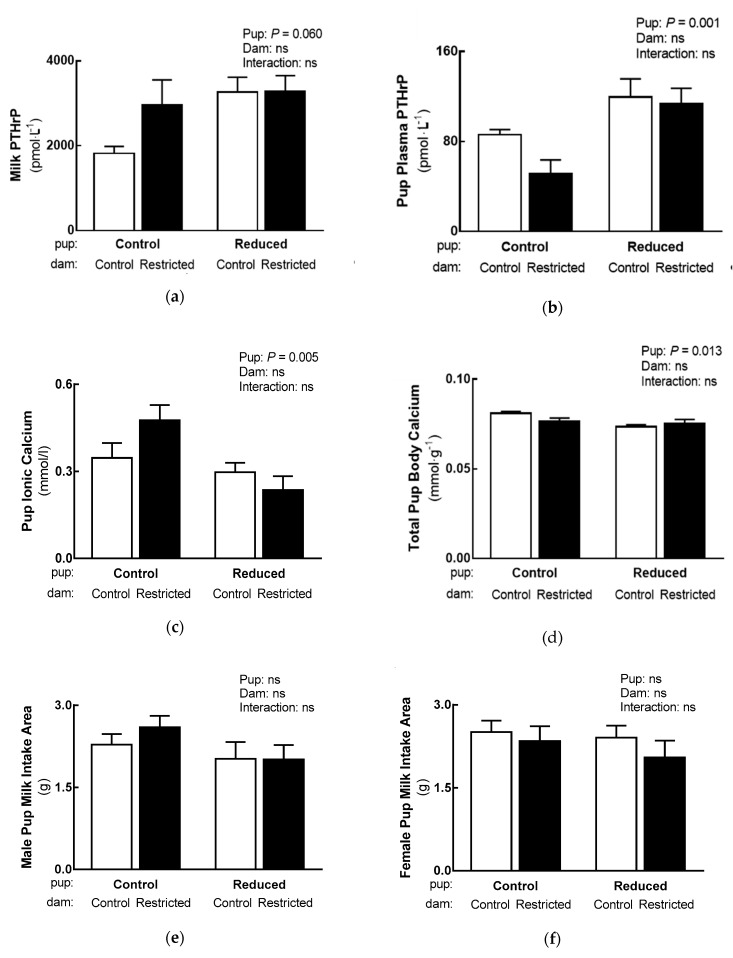
Effect reducing litter size has on pup calcium handling and milk intake (*n* = 6–10 litter averages per group, where appropriate). (**a**) Milk and (**b**) pup PTHrP concentrations, (**c**) pup ionic calcium concentrations, (**d**) pup total body calcium and (**e**,**f**) pup milk intake. Data are analysed with a two-way ANOVA reporting differences between Pup (Standard and Reduced litters) and Dam (Normal and Altered milk quality/composition) groups. Data presented as the mean ± SEM, where ns is not significant. Normal milk quality/composition dams denoted by white open bars and Altered milk quality/composition dams denoted by black closed bars.

Milk PTHrP tended to be increased in dams suckling Reduced litter size pups (Figure 3a; *p* = 0.060, two-way ANOVA). Milk Na^+^/K^+^, ionic and total calcium, total protein and lactose were not affected by maternal milk quality/composition or litter size (Table 2). A main Pup effect was identified in pup plasma PTHrP, where it was increased in Reduced litter size pups (+69%; Figure 3b). This finding was in conjunction with main Pup effects in pup ionic calcium and total body calcium, where they were decreased in Reduced litter size pups (−35% and −5%, respectively; Figure 3c,d). Milk intake in both male and female pups were not affected by maternal milk quality/composition or pup litter size (Figure 3e,f).

### 3.4. Milk Fatty Acid Composition

An interaction between Dam and Pup was identified in linoleic acid (LA), arachidonic acid (AA) and total n-6 fatty acids. Specifically, levels of the individual n-6 PUFAs (LA and AA), and total n-6 fatty acid content were increased in milk from Standard litter size pups suckled by Altered milk quality/composition dams (+20% and +21% for LA and total n-6) and Reduced litter size pups suckled by Normal milk quality/composition(+21%, +48% and +24%, respectively) dams compared to Standard litter size pups suckled by Normal milk quality/composition dams (Table 3; Tukey’s post-hoc). There was a main Pup effect in levels of the n-3 LC-PUFA docosahexaenoic acid (DHA) which was increased in milk from dams suckling Reduced litter size pups (+31%; Table 3), but there was no difference in total n-3 fatty acid content of the milk between groups. An interaction between Dam and Pup was identified in long chain saturated fatty acid content, where they were decreased in the milk of Reduced litter size pups suckled by Normal milk quality/composition dams compared to Standard litter size pups suckled by Normal milk quality/composition dams (−24%; Table 3, Tukey’s post-hoc). A main Pup effect was identified in total LC-MUFA content, where it was increased in the milk of dams suckling Reduced litter pups (+11%). The ratio of n-6:n-3 fatty acids and medium saturated fatty acids were not affected by litter size or maternal milk quality/composition (Table 3). The full list of fatty acids analyzed from the stomach contents are reported in Supplementary Table S1.

### 3.5. Adult Health

**Figure 4:**
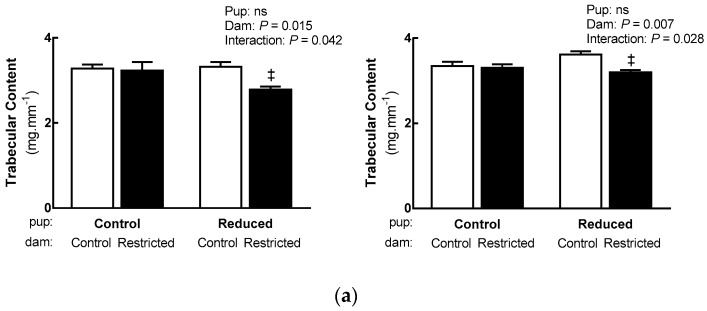

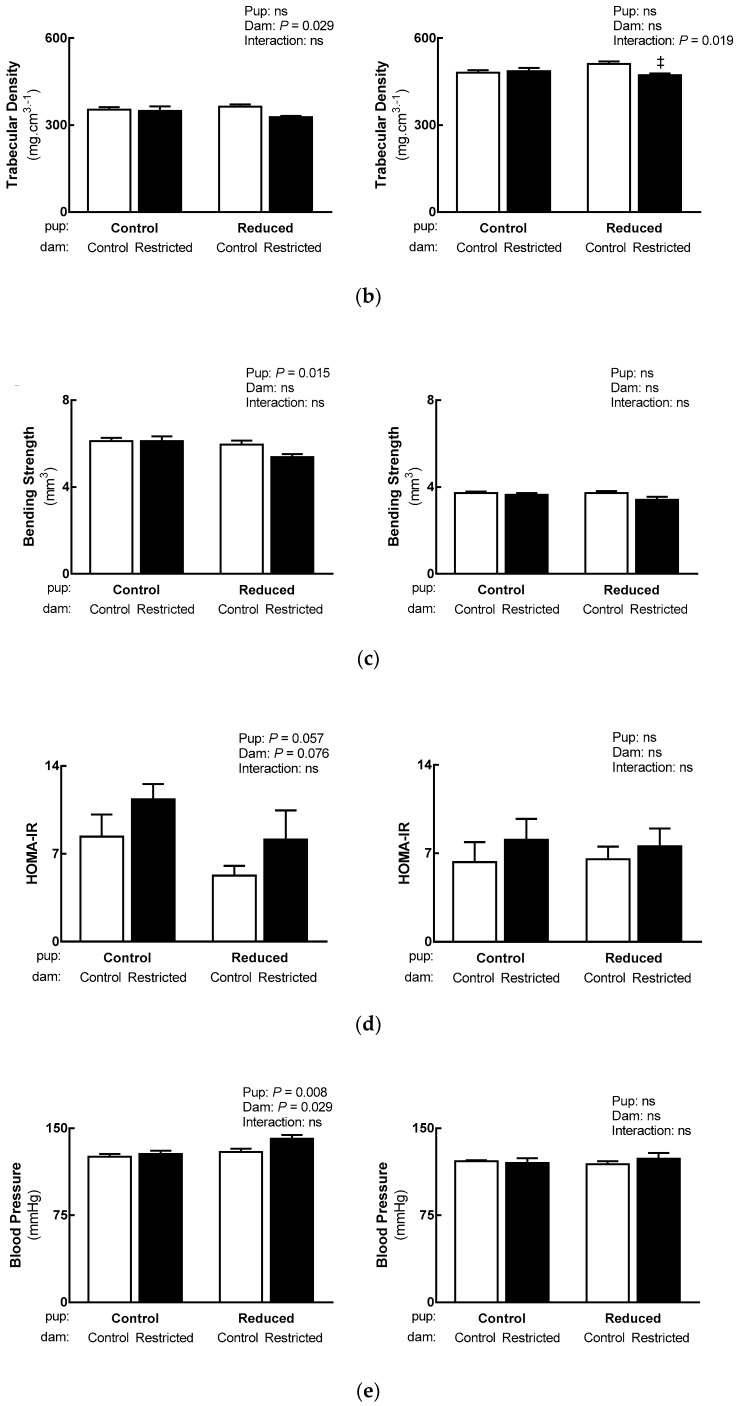
Effect reducing litter size has on adult offspring bone (*n* = 9–19 per group, with *n* = 1 representing 1 male and female per litter) and cardiometabolic health (*n* = 5–11 per group, with *n* = 1 representing 1 male and female per litter). (**a**,**b**) Trabecular mineral content and density, (**c**) bone bending strength, (**d**) homeostasis model assessment for insulin resistance (HOMA-IR) and (**e**) blood pressure at 6 months. Data are analysed with a two-way ANOVA reporting differences between Pup (Standard and Reduced litters) and Dam (Normal and Altered milk quality/composition) groups, with a Tukey’s post-hoc test used to identify where interactions lie. Data presented as the mean ± SEM, where ns is not significant. Significant differences between sham operated (Control; Normal milk quality/composition) and uteroplacental insufficiency surgery (Restricted; Altered milk quality/composition) dams are indicated with a double dagger (‡ *p* < 0.05). Normal milk quality/composition dams denoted by white open bars and Altered milk quality/composition dams denoted by black closed bars, with data from males on the left-hand side and data from females on the right-hand side.

Body weight at 6 months was decreased in male (−5%), but not female, offspring from Reduced litters, which was further exaggerated by Altered milk quality/composition (−5%) (Table 4). Femur length in both males and females was unaffected by litter size or maternal milk quality/composition (Table 4). An interaction between Pup and Dam was identified in male and female trabecular mineral content, whereby trabecular mineral content was decreased in male (−14%) and female (−12%) Reduced litter size pups suckled by Altered milk quality/composition dams compared to Reduced litter size pups suckled by Normal milk quality/composition counterparts (Figure 4a; Tukey’s post-hoc). A main Dam effect was identified in trabecular density in male offspring, where it decreased in offspring suckled by Altered milk quality/composition dams (−6%; Figure 4b). In females, an interaction between Pup and Dam was identified trabecular density, where it was decreased in Reduced litter size pups suckled by Altered milk quality/composition dams (−8%) compared to Reduced litter size pups suckled by Normal milk quality/composition dams (Figure 4b; Tukey’s post-hoc). Cortical mineral content was decreased in male offspring who were suckled by Altered milk quality/composition dams (−4%), with no change in females (Table 4). Cortical density was unaffected by litter size or maternal milk quality/composition in both male and female offspring (Table 4). A main Pup effect was identified in bone bending strength in male, but not female offspring, where it was decreased in offspring of Reduced litters (−7%; Figure 4c).

Fasting glucose at 6 months was not different between groups in either males or females (Table 4). In male, but not female offspring, a main Dam effect was identified in fasting insulin concentrations where it was higher in offspring who had been suckled by Altered milk quality/composition dams and also tended to be lower in Reduced litter size pups (Table 4; *p* = 0.070). Similarly, glucose AUC tended to be higher and first phase insulin tended to be lower in male offspring that were suckling Altered milk quality/composition dams (Table 4; *p* = 0.080 and *p* = 0.069, respectively), with no changes in females. HOMA-IR at 6 months tended to be increased in offspring who had been suckled by Altered milk quality/composition dams (*p* = 0.076) and decreased in Reduced litter size offspring (*p* = 0.057; Figure 4d). Interestingly, a main Pup and Dam effect were observed in male blood pressure, where it was increased both in Reduced litter male offspring compared to Standard litter size offspring (+7%) and in offspring who had been suckled by Altered milk quality/composition dams compared to Normal milk quality/composition dams (+5%; Figure 4e). HOMA-IR and blood pressure were not different between groups in female offspring (Figure 4d,e).

## 4. Discussion

Providing offspring with adequate and appropriate nutrition during lactation is essential for long-term health, with both over- and undernutrition increasing disease susceptibility [14,15,16]. We have previously demonstrated that both the dam and suckling pup can influence maternal milk composition, and that these effects generally represent an attempt to improve pup growth and development [34]. The current study has demonstrated that reducing litter size at birth, regardless of whether the dam has normal or altered milk quality/composition impairs pup calcium homeostasis, which may reduce bone bending strength in male offspring. Additionally, these adult Reduced litter males appear to have increased cardiovascular disease risk and decreased diabetes risk. Furthermore, altered milk quality/composition (induced by uteroplacental insufficiency surgery) similarly programs poor bone health, high blood pressure and impairs glucose tolerance/insulin sensitivity. These finding are likely due to differential influences of the predetermined litter size at birth [26] and effects on milk quality. This highlights that caution needs to be taken when interpreting animal studies that focus on early life nutrition, as the experimental approach may independently modulate milk nutrition and quality.

### 4.1. Effects of Reducing Litter Size

It is important to note that as all dams underwent a surgical procedure (sham or ligation) and all litters were cross-fostered the effects reported are as direct consequences of either the maternal surgery (resulting in altered milk quality/composition) or reduced litter size, thus we have adequately accounted for all possible confounders, such as stress, across experimental groups. Additionally, growth restricted pups were not included in the study, only pups born from sham-operated dams that have intact or reduced litter sizes when cross-fostered, which would limit any changes observed in pups suckled by dams that underwent uteroplacental insufficiency surgery to the lactation environment they are exposed to in early postnatal life. Reducing litter size is commonly used in the developmental programming field, however recent studies demonstrate that this can independently program disease susceptibility [27,28,29,30]. In the current study we report dynamic changes in pup calcium homeostasis and maternal milk composition as a result of reducing the litter size to 5 pups. Interestingly, despite these changes in milk composition, no changes in postnatal body weight was observed, which suggests that other intrinsic hormonal factors may be responsible for maintaining pup growth that requires further studies. The reduction in mammary gland weight in Normal milk quality/composition dams suckled by Reduced litter pups may be due to the decreased *b-casein* gene expression, which is known to regulate mammary differentiation and is a regulator of milk protein gene expression [49]. However, this likely did not alter alveolar area or number as lactogenic differentiation is completed by PN6 [49]; although studies at earlier postnatal ages may reveal altered mammary structure. Despite no changes in maternal PTHrP, dams suckled by Reduced litter pups had increased milk PTHrP. Previous studies have demonstrated that milk PTHrP is not absorbed by the intestines into the pups circulation [50], suggesting that the increased pup PTHrP concentrations we report are independent of milk PTHrP content. This increased pup PTHrP is likely to stimulate bone resorption, and thus calcium release, in an attempt to increase plasma calcium concentrations, as shown in a previous study [51], but has also been shown to result in decreased postnatal body calcium content [52]. Interestingly, the increased maternal ionic calcium concentrations in Normal milk quality/composition dams suckled by Reduced litter pups are likely an attempt to increase milk calcium concentrations to compensate for this deficit. This, however, did not translate to increased milk calcium content at the time point investigated in this study, highlighting that milk composition needs to be assessed at additional postnatal ages. Nevertheless, the increased milk PTHrP in dams suckling Reduced litter pups would facilitate increased intestinal calcium reabsorption to increase pup plasma calcium concentrations, preventing any further bone breakdown. These data may highlight why the Reduced litter size pups suckled by Normal milk quality/quantity dams do not exhibit the same deficits in adult bone mineral content and density that are observed in the Reduced litter size pups suckled by Altered milk quality/quantity. Despite this, however, Reduced litter male offspring have decreased bending strength, indicative of increased fracture risk and thus poor bone health, suggesting that any compensatory changes in milk composition were not sufficient to fully prevent bone deficits.

In addition to alterations in pup calcium homeostasis in the Reduced litter group, we also report changes in milk fatty acid content, highlighting that Reducing litter size has profound effects on maternal milk quality. Interestingly, milk n-6 fatty acid content was increased in the Reduced litter size pups suckled by Normal milk quality/quantity dams. This is significant, since increased n-6 fatty acid intakes have been associated with increased fat deposition in early life [11]. High intakes of n-6 PUFA during lactation also influence lipid tissue status and modulate metabolic pathways that can lead to diabetes and cardiovascular disease [11]. It is important to note, however, that it is the balance of omega-6 and omega-3 PUFA in the diet that appears to be the more important determinant of physiological effects than levels of either PUFA type alone. In the current study, the n-6:n-3 ratio was not altered, at least at this age, by either litter size or maternal uteroplacental insufficiency surgery. This suggests that the observed changes in milk PUFA composition may not be a major factor contributing to the male onset cardiovascular disease we report, but may instead be due to the decreased post-weaning growth trajectory. Interestingly, reducing litter size increased milk LC-MUFA and DHA (Reduced litter size pups suckled by Normal milk quality/quantity dams only) content, which is known to have beneficial effects on metabolic function during critical periods of development [11]. The improved metabolic health in Reduced litter size pups suckled by Normal milk quality/quantity dams male offspring may also be attributed to increased milk Pentadecanoic acid (15:0) and reduced Palmitic acid (16:0) both of which are known to reduce the risk of type 2 diabetes [53]. This may explain why the Reduced litter size male offspring appear to have a decreased risk of developing diabetes.

### 4.2. Effects of Altered Milk Quality/Composition

Surprisingly alterations in milk quality/composition, induced by maternal uteroplacental insufficiency surgery, resulted in very few changes in milk composition compared to the large number of changes associated with reducing litter size and slowing offspring postnatal growth. In the current study, we report that dams with altered milk quality/composition have decreased mammary *a-lactalbumin* gene abundance. If this translates to decreased milk α-lactalbumin protein then this may, at least in part, contribute to the poor adult bone health we observed in male offspring reared by Altered milk quality/composition dams. Specifically, as α-lactalbumin binds calcium [54] it is possible that decreased milk α-lactalbumin impairs calcium delivery to the pup, compromising bone mineralization and development. This poor bone health was only apparent in Reduced litter size pups suckled by Altered milk quality/composition dams, likely because the effect was compounded by the decreased maternal ionic concentrations that may have compromised calcium delivery at later lactational ages. Interestingly, a recent study demonstrated that supplementing 6-week old obese diabetic Zucker rats with α-lactalbumin for 13 weeks improves metabolic function [55]; highlighting the benefits of α-lactalbumin on metabolic health. If the inverse is also true, and rats are exposed to decreased α-lactalbumin during development, it is possible that this may contribute to the increased risk of adult metabolic disease we observed in our rats. In addition, the increased n-6 fatty acid intake in Standard litter size pups suckled by Altered milk quality/quantity dams during lactation may also contribute to the increased blood pressure and impaired insulin sensitivity in male offspring [11]. As the female offspring suckled by Altered milk quality/composition dams caught up in body weight prior to weaning they may be protected against developing cardiometabolic disease, due to the benefits of early accelerated growth for long-term cardiometabolic health [36,56,57,58].

### 4.3. Study Limitations

Despite our study demonstrating that reducing the litter size from 9 pups to 5 pups alters milk quality/composition and has implications for long-term offspring health, further studies are required to investigate the effects of the more common practice of reducing litter sizes to 8–10 pups from original litter sizes of 12–20 pups, since this also has the potential to influence milk quality/quantity and thus offspring outcomes. Nevertheless, the findings of the current study highlight the potential impact reducing litter size, independent of other neonatal factors, has on programming offspring outcomes. An important factor that was not taken into consideration in the present study is the impact of offspring sex on milk composition, due to difficulties in controlling for this in our large litter bearing animal model. Indeed, several epidemiological and experimental (with smaller litter bearing animals) studies have well demonstrated differences in milk composition between male and female infants [59,60]; demonstrating that infant sex has a significant impact on milk quality. This highlights the need for well-controlled human and animal studies to identify alterations in milk composition between infant sexes in several different pregnancy complications.

A limitation of the current study is that maternal behavior was not evaluated throughout lactation. This is particularly important, as the quality and quantity of maternal care during lactation can impact on offspring behavioral, endocrine and neural development (see review by Curley and Champagne [61]). Specifically, a recent study demonstrated that dams whose litters were reduced to 3 pups (to induce postnatal overnutrition) have improved maternal care, characterize by increased time devoted to arched nursing and licking pups [62]. Not surprisingly the pups had early accelerated growth and were overweight by PN60, as indicated by increased adiposity, and were hyperglycemic and hyperleptinemic [62]. These findings are however difficult to compare directly to the results of the current study where the Reduced litter pups had decreased postnatal growth, which suggests that the extent of litter size reduction in the current study is unlikely to have resulted in improved maternal care.

## 5. Conclusions

This study demonstrates that reducing pup litter size and altered maternal milk quality/composition differentially program poor adult offspring health, which is likely due to altered milk quality. Specifically, reducing the litter size alters milk composition, impairs pup calcium homeostasis and programs poor bone health, which likely contributes to the lower diabetes risk and poor cardiovascular health. Alterations in maternal milk quality/composition, on the other hand, programs poor adult bone and cardiovascular health and reduces glucose tolerance/insulin sensitivity. This study highlights the need for appropriate controls in developmental research to clearly ascertain phenotypes in the model. Importantly, controls implemented to standardize outcomes across treatment groups may independently program disease susceptibility, which limit their translatability. Therefore, care must be taken in interpreting findings from studies that standardize litter size as it may mask subtle effects on cardiometabolic health.

## Figures and Tables

**Table 1:** Cross-fostered litter size at postnatal day (PN) 6 and body weight from birth (PN1) to weaning (PN35) (*n* = 12–18 litter averages per group).

Pup-on-Dam		Standard-on-Normal	Standard-on-Altered	Reduced-on-Normal	Reduced-on-Altered	Two-Way ANOVA		
						Pup	Dam	Interaction
**Litter Size**								
PN1		10.4 ± 0.4	11.5 ± 0.9	5.0 ± 0.0	6.0 ± 0.7	*p* = 0.0001	ns ^1^	ns
PN6		8.9 ± 0.6	8.6 ± 0.8	4.7 ± 0.3	5.25 ± 0.8	*p* = 0.0001	ns	ns
Cannibalism		1.5 ± 0.5	2.9 ± 1.0	1.0 ± 0.7	0.8 ± 0.3	ns	ns	ns
**Body Weight (g)**								
PN1	Male	4.17 ± 0.12	4.13 ± 0.10	4.23 ± 0.15	3.99 ± 0.13	ns	ns	ns
	Female	3.87 ± 0.12	3.98 ± 0.06	3.98 ± 0.06	3.94 ± 0.11	ns	ns	ns
PN3	Male	5.62 ± 0.11	5.42 ± 0.15	5.41 ± 0.14	5.12 ± 0.15	ns	ns	ns
	Female	5.38 ± 0.12	5.16 ± 0.20	5.23 ± 0.14	4.91 ± 0.14	ns	ns	ns
PN6	Male	8.54 ± 0.21	8.31 ± 0.23	8.66 ± 0.38	7.65 ± 0.30	ns	*p* = 0.039	ns
	Female	8.17 ± 0.21	7.91 ± 0.37	8.29 ± 5.14	7.26 ± 0.28	ns	*p* = 0.049	ns
PN10	Male	14.6 ± 0.3	14.1 ± 0.4	14.6 ± 0.5	13.1 ± 0.5	ns	*p* = 0.037	ns
	Female	14.2 ± 0.3	13.6 ± 0.6	14.1 ± 0.5	12.7 ± 0.5	ns	*p* = 0.040	ns
PN14	Male	22.1 ± 0.5	21.2 ± 0.6	21.5 ± 0.7	19.6 ± 1.0	ns	*p* = 0.043	ns
	Female	21.7 ± 0.4	20.6 ± 0.8	20.6 ± 0.8	19.0 ± 0.9	ns	ns	ns
PN17	Male	27.4 ± 0.6	26.3 ± 0.6	26.9 ± 0.8	24.5 ± 1.2	ns	*p* = 0.030	ns
	Female	26.9 ± 0.5	25.6 ± 0.7	26.0 ± 0.8	24.1 ± 1.1	ns	*p* = 0.047	ns
PN21	Male	34.3 ± 0.6	33.6 ± 1.0	34.7 ± 0.7	31.4 ± 1.1	ns	*p* = 0.020	ns
	Female	33.8 ± 0.6	32.8 ± 1.0	33.5 ± 0.9	31.4 ± 1.0	ns	ns	ns
PN24	Male	43.7 ± 1.1	42.3 ± 0.9	44.3 ± 1.0	39.2 ± 1.4	ns	*p* = 0.004	ns
	Female	42.1 ± 0.8	40.4 ± 1.1	42.4 ± 1.1	38.5 ± 1.2	ns	*p* = 0.010	ns
PN28	Male	58.7 ± 1.2	57.4 ± 1.1	59.6 ± 1.2	54.0 ± 1.8	ns	*p* = 0.011	ns
	Female	54.9 ± 1.0	54.1 ± 1.4	55.0 ± 1.4	51.3 ± 1.3	ns	ns	ns
PN35	Male	87.7 ± 1.7	86.6 ± 1.7	87.1 ± 1.3	82.8 ± 2.8	ns	ns	ns
	Female	79.1 ± 1.2	77.8 ± 1.8	77.0 ± 1.4	75.7 ± 1.8	ns	ns	ns

Data are analysed with a two-way ANOVA reporting differences between Pup (Standard and Reduced litters) and Dam (Normal and Altered milk quality/composition) groups. Data presented as the mean ± SEM, where ns is not significant. ^1^ ns is not significant.

**Table 2:** Maternal plasma, mammary and pooled milk composition in the four cross-foster groups on postnatal day 6.

Pup-on-Dam	Standard-on-Normal	Standard-on-Altered	Reduced-on-Normal	Reduced-on-Altered	Two-Way ANOVA		
					Pup	Dam	Interaction
**Maternal (*n* = 4–9 per group)**							
Plasma PTHrP (pmol/L)	8.2 ± 0.4	9.1 ± 1.2	10.5 ± 0.8	9.6 ± 0.7	ns ^1^	ns	ns
Plasma total calcium (mmol/L)	2.6 ± 0.08	2.6 ± 0.04	2.6 ± 0.05	2.4 ± 0.04	ns	ns	ns
Plasma corticosterone (ng/mL)	593 ± 59	726 ± 106	551 ± 86	553 ± 80	ns	ns	ns
**Mammary (*n* = 3–6 per group)**							
Alveolar number	14.5 ± 1.6	15.9 ± 0.8	11.4 ± 1.2	16.5 ± 1.7	ns	*p* = 0.038	ns
Alveolar area	75.2 ± 3.0	75.1 ± 1.9	79.8 ± 6.5	78.5 ± 3.9	ns	ns	ns
PTHrP mRNA	1.0 ± 0.1	0.8 ± 0.3	1.1 ± 0.2	1.4 ± 0.3	ns	ns	ns
**Milk (*n* = 4–8 per group)**							
Na/K	7.6 ± 1.2	6.5 ± 1.6	9.8 ± 2.5	6.4 ± 0.9	ns	ns	ns
Ionic calcium (mmol/L)	8.9 ± 0.6	8.7 ± 1.0	7.4 ± 0.9	9.1 ± 0.4	ns	ns	ns
Total calcium (mmol/L)	64.7 ± 4.0	65.8 ± 3.7	60.4 ± 5.6	57.9 ± 2.8	ns	ns	ns
Total protein (mg/L)	28.2 ± 4.4	22.0 ± 3.7	25.4 ± 4.5	28.1 ± 3.6	ns	ns	ns
Lactose (mM)	30.2 ± 7.9	38.4 ± 3.8	29.5 ± 6.0	41.7 ± 10.4	ns	ns	ns

Data are analysed with a two-way ANOVA reporting differences between Pup (Standard and Reduced litters) and Dam (Normal and Altered milk quality/composition) groups. Data presented as the mean ± SEM with sex pooled per litter, where ns is not significant. ^1^ ns is not significant.

**Table 3:** Fatty acid composition in stomach contents consumed in milk presented as a cumulative total percentage of fatty acids on postnatal day 6 in the four cross-foster groups (*n* = 5–7 per group, with *n* = 1 representing data from 1 pooled litter).

Pup-on-Dam	Standard-on-Normal	Standard-on-Altered	Reduced-on-Normal	Reduced-on-Altered	Two-Way ANOVA		
					Pup	Dam	Interaction
**Fatty Acids**							
*Omega-6 PUFA*							
Linoleic (18:2n-6)	11.79 ± 0.25	14.12 ± 0.43 ^‡1^	14.23 ± 0.26 *^2^	13.85 ± 0.50	*p* = 0.010	*p* = 0.019	*p* = 0.002
Arachidonic (20:4n-6)	1.03 ± 0.06	1.33 ± 0.12	1.52 ± 0.08 *	1.35 ± 0.09	*p* = 0.014	ns ^3^	*p* = 0.023
Total n-6 PUFA	14.4 ± 0.4	17.4 ± 0.7 ^‡^	17.9 ± 0.4 *	17.1 ± 0.7	*p* = 0.009	ns	*p* = 0.003
*Omega-3 PUFA*							
α-linolenic (18:3n-3)	1.40 ± 0.06	1.48 ± 0.09	1.49 ± 0.05	1.46 ± 0.11	ns	ns	ns
Eicosapentaenoic (20:5n-3)	0.21 ± 0.01	0.17 ± 0.03	0.21 ± 0.02	0.18 ± 0.04	ns	ns	ns
Docosahexaenoic (22:6n-3)	0.41 ± 0.03	0.45 ± 0.06	0.55 ± 0.04	0.58 ± 0.09	*p* = 0.047	ns	ns
Total n-3 PUFA	2.4 ± 0.09	2.4 ± 0.18	2.7 ± 0.15	2.7 ± 0.21	ns	ns	ns
n-6:n-3	6.07 ± 0.15	7.11 ± 0.27	6.58 ± 0.28	6.63 ± 0.54	ns	ns	ns
Total LC-MUFA	22.4 ± 0.9	22.4 ± 1.4	25.8 ± 0.9	23.9 ± 0.8	*p* = 0.032	ns	ns
**Saturated Fatty Acids**							
Medium (6–12)	19.8 ± 1.0	21.1 ± 0.7	21.1 ± 0.7	20.8 ± 0.9	ns	ns	ns
Long (14–20)	40.4 ± 1.1	35.2 ± 2.1	30.9 ± 1.4 ^*^	34.2 ± 1.9	*p* = 0.006	ns	*p* = 0.022

Data are analysed with a two-way ANOVA reporting differences between Pup (Standard and Reduced litters) and Dam (Normal and Altered milk quality/composition) groups, with a Tukey’s post-hoc test used to identify where interactions lie. Data presented as the mean ± SEM with sex pooled per litter, where ns is not significant. Significant differences between Standard and Reduced litter pups are indicated by an asterisk (* *p* < 0.05) and differences between sham operated (Control; Normal milk quality/composition) and uteroplacental insufficiency surgery (Restricted; Altered milk quality/composition) dams are indicated with a double dagger (^‡^ *p* < 0.05). Also see Table S1 for individual fatty acids. ^1^ *p* < 0.05 Standard-on-Altered vs. Standard-on-Normal; ^2^ *p* < 0.05 Reduced-on-Normal vs. Standard-on-Normal; ^3^ ns is not significant.

**Table 4:** Adult offspring physiology in the four cross-foster groups at 6 months (*n* = 9–19 per group for bone analyses and *n* = 5–11 per group for cardiometabolic analyses, with *n* = 1 representing 1 male and female per litter).

Pup-on-Dam		Standard-on-Normal	Standard-on-Altered	Reduced-on-Normal	Reduced-on-Altered	Two-Way ANOVA		
						Pup	Dam	Interaction
Body Weight (g)	Male	386.1 ± 7.0	368.9 ± 2.3	368.4 ± 7.0	351.8 ± 9.5	*p* = 0.020	*p* = 0.023	ns
	Female	239.3 ± 4.1	233.1 ± 6.5	238.3 ± 3.7	226.9 ± 4.7	ns ^1^	ns	ns
**Bone Parameters**								
Femur Length (mm)	Male	37.01 ± 0.12	36.79 ± 0.23	36.61 ± 0.21	36.01 ± 0.16	ns	ns	ns
	Female	33.09 ± 0.14	32.75 ± 0.19	32.69 ± 0.14	32.42 ± 0.15	ns	ns	ns
Cortical content (mg·mm^−1^)	Male	10.67 ± 0.15	10.63 ± 0.28	10.74 ± 0.18	9.93 ± 0.13	ns	*p* = 0.005	ns
	Female	7.87 ± 0.09	7.79 ± 0.11	7.90 ± 0.12	7.49 ± 0.11	ns	ns	ns
Cortical density (mg (mm^3^)^−1^)	Male	1409.4 ± 1.8	1413.3 ± 3.7	1412.6 ± 3.8	1407.9 ± 1.9	ns	ns	ns
	Female	1407.9 ± 2.5	1412.4 ± 1.2	1404.8 ± 1.8	1399.1 ± 2.0	ns	ns	ns
**Metabolic Parameters**								
Fasting plasma glucose (mmol·L^−1^)	Male	5.77 ± 0.21	5.81 ± 0.19	5.52 ± 0.19	5.64 ± 0.27	ns	ns	ns
	Female	5.15 ± 0.18	5.69 ± 0.18	5.50 ± 0.20	5.64 ± 0.22	ns	ns	ns
Fasting plasma insulin (ng·ML^−1^)	Male	1.28 ± 0.24	1.82 ± 0.17	0.91 ± 0.12	1.32 ± 0.33	*p* = 0.070	*p* = 0.048	ns
	Female	1.07 ± 0.24	1.23 ± 0.23	1.09 ± 0.14	1.23 ± 0.21	ns	ns	ns
Glucose AUC	Male	757.3 ± 87.0	795.6 ± 79.8	742.2 ± 67.1	989.6 ± 62.1	ns	*P* = 0.080	ns
	Female	812.9 ± 74.1	701.9 ± 70.1	689.3 ± 52.8	635.2 ± 45.2	ns	ns	ns
First phase insulin AUC	Male	14.25 ± 1.54	10.01 ± 2.08	11.31 ± 1.92	8.53 ± 1.17	ns	*p* = 0.069	ns
	Female	13.91 ± 2.02	11.29 ± 2.05	13.81 ± 1.74	14.01 ± 1.84	ns	ns	ns

Data are analysed with a two-way ANOVA reporting differences between Pup (Standard and Reduced litters) and Dam (Normal and Altered milk quality/composition) groups. Data presented as the mean ± SEM, where ns is not significant. ^1^ ns is not significant.

## Supplementary Materials

The following are available online at http://www.mdpi.com/2072-6643/11/1/118/s1, Table S1: Fatty acid composition in stomach contents on postnatal day 6.
