# Supplementary material for: Reducing Pup Litter Size Alters Early Postnatal Calcium Homeostasis and Programs Adverse Adult Cardiovascular and Bone Health in Male Rats
Source: Nutrients. 2019 Jan 8;11(1):118. doi: 10.3390/nu11010118 (PMC6356436; doi:10.3390/nu11010118)
Supplement: Supplementary file 1 [file nutrients-11-00118-s001.pdf]

Table S1 – related to Table 3. Fatty acid composition in stomach contents consumed in milk as a percentage of total of total fatty acids on postnatal day 6 (n = 6-7 per group, with n = 1 representing data from 1 pooled litter). Data are analysed with a two-way ANOVA reporting differences between Pup (Standard and Reduced litters) and Dam (Normal and Altered milk quality/composition) groups, with a Tukey's post-hoc used to identify where interactions lie. Data presented as the mean ± SEM, where ns is not significant. Significant differences between Standard and Reduced litter pups are indicated by an asterisk (*$P < 0.05$) and differences between sham operated (Control; Normal milk quality/composition) and uteroplacental insufficiency surgery (Restricted; Altered milk quality/composition) dams are indicated with a double dagger (‡ $p < 0.05$).

**Pup -on- Dam**	**Standard -on- Normal**	**Standard -on- Altered**	**Reduced -on- Normal**	**Reduced -on- Altered**	**Two-way ANOVA**		
					**Pup**	**Dam**	**Interaction**
**Saturated fatty acids (%)**							
Medium chain (%)							
8:0 Caprylic	1.15±0.21	1.05±0.19	2.08±0.23	1.44±0.26	$p = 0.008$	ns	ns
10:0 Capric	8.61±0.48	8.44±1.43	9.90±0.34	9.65±0.54	ns	ns	ns
12:0 Lauric	10.07±0.34	8.74±1.50	9.16±0.32	9.76±0.30	ns	ns	ns
Long chain (%)							
14:0 Myristic	12.44±0.44	11.03±0.89	9.01±0.65	10.28±0.70	$p = 0.008$	ns	ns
15:0 Pentadecanoic	0.17±0.01	0.24±0.01‡	0.20±0.01*	0.21±0.01	ns	$p = 0.001$	$p = 0.004$
16:0 Palmitic	24.34±0.76	21.06±1.24	18.90±0.75*	20.70±1.22	$p = 0.012$	ns	$p = 0.025$
17:0 Margaric	0.18±0.01	0.17±0.03	0.17±0.01	0.15±0.03	ns	ns	ns
18:0 Stearic	3.14±0.24	2.57±0.04‡	2.57±0.07*	2.76±0.08	ns	ns	$p = 0.007$
20:0 Arachidic	0.10±0.02	0.09±0.02	0.08±0.02	0.10±0.02	ns	ns	ns
Monounsaturated fatty acids (%)							
12:1 Lauroleic	0.00±0.00	0.28±0.11	0.57±0.24	0.57±0.21	$p = 0.016$	ns	ns
16:1 Palmitoleic	0.93±0.07	0.97±0.08	1.22±0.08	1.00±0.09	ns	ns	ns
17:1 Heptadecenoic	0.05±0.03	0.10±0.02	0.08±0.02	0.08±0.03	ns	ns	ns
18:1n-9 Oleic	20.87±0.82	20.58±1.24	23.29±0.88	21.69±0.66	ns	ns	ns
20:1n-9 Gondoic	0.51±0.03	0.50±0.04	0.59±0.03	0.54±0.02	ns	ns	ns
LC-PUFA (n-6) (%)							
18:3n-6 γ-linolenic	0.20±0.01	0.25±0.01‡	0.26±0.01*	0.23±0.02	ns	ns	$p = 0.006$
20:2 Eciosatrienoic	0.53±0.02	0.58±0.03	0.63±0.03	0.60±0.03	$p = 0.051$	ns	ns
20:3n-6 Dihomo-γ-linolenic	0.35±0.02	0.40±0.02	0.40±0.01	0.39±0.01	ns	ns	ns
22:4n-6 Docosatetraenoic	0.49±0.04	0.69±0.10	0.82±0.07*	0.70±0.06	$p = 0.029$	ns	$p = 0.046$
LC-PUFA (n-3) (%)							
22:5n-3 Docosapentaenoic	0.36±0.03	0.38±0.04	0.50±0.04	0.43±0.03	$p = 0.016$	ns	ns
